# Crystal Structure of UBA2^ufd^-Ubc9: Insights into E1-E2 Interactions in Sumo Pathways

**DOI:** 10.1371/journal.pone.0015805

**Published:** 2010-12-30

**Authors:** Jing Wang, Asad M. Taherbhoy, Harold W. Hunt, Steven N. Seyedin, David W. Miller, Darcie J. Miller, Danny T. Huang, Brenda A. Schulman

**Affiliations:** 1 Department of Structural Biology, St. Jude Children's Research Hospital, Memphis, Tennessee, United States of America; 2 Integrated Program in Biomedical Sciences, University of Tennessee Health Science Center, Memphis, Tennessee, United States of America; 3 Howard Hughes Medical Institute, St. Jude Children's Research Hospital, Memphis, Tennessee, United States of America; University of Queensland, Australia

## Abstract

Canonical ubiquitin-like proteins (UBLs) such as ubiquitin, Sumo, NEDD8, and ISG15 are ligated to targets by E1-E2-E3 multienzyme cascades. The Sumo cascade, conserved among all eukaryotes, regulates numerous biological processes including protein localization, transcription, DNA replication, and mitosis. Sumo conjugation is initiated by the heterodimeric Aos1-Uba2 E1 enzyme (in humans called Sae1-Uba2), which activates Sumo's C-terminus, binds the dedicated E2 enzyme Ubc9, and promotes Sumo C-terminal transfer between the Uba2 and Ubc9 catalytic cysteines. To gain insights into details of E1-E2 interactions in the Sumo pathway, we determined crystal structures of the C-terminal ubiquitin fold domain (ufd) from yeast Uba2 (Uba2^ufd^), alone and in complex with Ubc9. The overall structures of both yeast Uba2^ufd^ and Ubc9 superimpose well on their individual human counterparts, suggesting conservation of fundamental features of Sumo conjugation. Docking the Uba2^ufd^-Ubc9 and prior full-length human Uba2 structures allows generation of models for steps in Sumo transfer from Uba2 to Ubc9, and supports the notion that Uba2 undergoes remarkable conformational changes during the reaction. Comparisons to previous structures from the NEDD8 cascade demonstrate that UBL cascades generally utilize some parallel E1-E2 interaction surfaces. In addition, the structure of the Uba2^ufd^-Ubc9 complex reveals interactions unique to Sumo E1 and E2. Comparison with a previous Ubc9-E3 complex structure demonstrates overlap between Uba2 and E3 binding sites on Ubc9, indicating that loading with Sumo and E3-catalyzed transfer to substrates are strictly separate steps. The results suggest mechanisms establishing specificity and order in Sumo conjugation cascades.

## Introduction

Post-translational modification by ubiquitin-like proteins (UBLs) is a major mechanism for regulating eukaryotic protein functions. UBLs generally become covalently attached to specific targets through a series of molecular “handoffs” involving multienzyme cascades consisting of an E1 activating enzyme, an E2 conjugating enzyme, and an E3 ligase (reviewed in [1]). Thus, it is of great interest to understand how E1, E2, and E3 enzymes interact with each other for UBL transfer.

Like other ubiquitin-like proteins (UBLs), the **S**mall **u**biquitin-related **mo**difier (Sumo) proteins become covalently ligated to targets (reviewed in [2], [3], [4]). Attachment of Sumo family UBLs are known to alter target functions such as protein-protein interactions, protein-DNA interactions, and subcellular localization (reviewed in [5]). As such, Sumo regulates many important processes, such as signaling, transcription, DNA repair and other stress responses, the cell cycle, and apoptosis [2], [3], [4]. Indeed, the budding yeast Smt3 protein (for simplification referred to as Sumo hereafter) regulates chromosome segregation, formation of the septin ring, and many other aspects of cell division [6], [7], [8].

Sumo family members are ligated to proteins via specific E1, E2, and E3 enzymes. The Sumo-specific E1 enzyme [the heterodimeric complex between Uba2 and Aos1 (yeast; termed Sae1-Uba2 in mammals)] initiates the process by first catalyzing adenylation of the Sumo C-terminus, which next becomes linked by a thioester bond to Uba2's catalytic cysteine [9], [10], [11], [12]. A transthiolation reaction ensues during which Sumo is transferred from Uba2 to the catalytic cysteine of the dedicated Sumo E2 conjugating enzyme, Ubc9 [13], [14], [15], [16]. Ultimately, either with or without facilitation by a Sumo-pathway-specific E3, Sumo is transferred from the Ubc9 catalytic cysteine to a target lysine ([17] and references therein). In some cases, repeated cycles of Sumo transfer lead to generation of polySumo chains on targets (for review, see [18]). In recent years, structural studies have provided details for many aspects of Sumo E1-E2-E3 conjugation cascades. These include structural understandings of how human Sumo is recognized and activated by Sae1-Uba2 [19], [20], how Ubc9 recognizes targets [17], [21], [22], [23], and how two distinctive E3s function in Sumo ligation [24], [25], [26]. Nonetheless, many fundamental aspects of the Sumo cascade remain incompletely understood.

For example, how does the Sumo E1 bind Ubc9? The structure of the human heterodimeric Sae1-Uba2 complex that comprises the Sumo E1 [19] displays conserved E1 domains [27], [28]: a heterodimeric “adenylation” domain comprised of portions of both Sae1 and Uba2, a domain of Uba2 containing the E1 catalytic cysteine, and a C-terminal ubiquitin-fold domain (ufd) from Uba2 that structurally resembles ubiquitin and Sumo [29]. For human Uba2, the catalytic cysteine domain has been shown to make weak interactions with Ubc9 [30]. In addition, for both yeast and human Uba2, the ufd has been implicated in Ubc9 recruitment.

For canonical UBL pathways, E2s generally appear to bind an E1 ufd [29]. Indeed, crystal structures from the pathway of another UBL, NEDD8, showed previously that the NEDD8 E1's ufd binds NEDD8 E2s, either Ubc12 (also known as UBE2M) or UBE2F, via the E2 N-terminal helix and β1β2-loop [31], [32], [33]. These E1^ufd^-E2 interactions appear to be conserved across the Sumo, NEDD8, ISG15 and ubiquitin pathways [29]. For example, replacing the UBA6 or UBA7 ufds with that of UBA1 was sufficient to swap E2 specificities, and substituting the E2 UbcH7's N-terminal helix and β1β2-loop regions with that of the E2 UbcH8 swaps E1 specificity [34], [35]. In terms of the Sumo pathway, the human Uba2 ufd was also shown to bind Ubc9 and to play an essential role in human Sae1-Sae2-mediated Sumo transfer to Ubc9 [19], [36]. The ufd of yeast Uba2 is essential for viability [19]. And from the E2 side, budding yeast Ubc9's N-terminal helix and β1β2-loop were also shown to be important for binding to Uba2 and formation of a Ubc9∼Sumo complex [37]. Notably, the Ubc9 β1β2-loop is significantly extended relative to other E2s, and has long been recognized as a unique structural feature of Ubc9 [38], [39]. Nonetheless, there is no high-resolution data for E1^ufd^-E2 interactions other than those for the NEDD8 pathway, despite their importance for the Sumo cascade in particular and UBL transfer in general. Therefore, to obtain a more detailed understanding of Uba2's ufd interactions with Ubc9 and to obtain broad insights into how E1-E2 specificity is established in general, we performed structural analysis of the Uba2^ufd^-Ubc9 complex from *S. cerevisiae*.

## Results and Discussion

### Structure of an isolated ubiquitin-fold domain from Uba2 (Uba2^ufd^)

**Figure 1:**
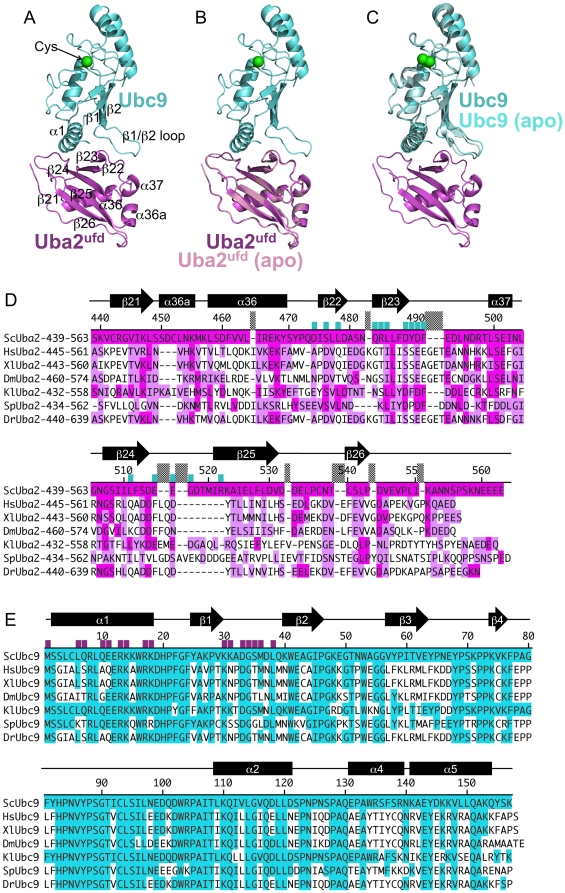
Overall structure of the Uba2^ufd^-Ubc9 complex. (A) Cartoon view of the overall structure of the complex with secondary structures numbered as previously described [19], [38], [39]. Uba2^ufd^ is shown in magenta. Ubc9 is shown in cyan. (B) Superposition of free Uba2^ufd^ (pink) superimposed with the Uba2^ufd^-Ubc9 complex. (C) Superposition of free Ubc9 (seafoam) [40] with the Uba2^ufd^-Ubc9 complex. (D) Sequence alignment of *Saccharomyces cerevisiae* Uba2^ufd^ sequence (Sc), with the corresponding regions of Uba2 from human (Hs), *Xenopus laevis* (Xl), *Drosophila melanogaster* (Dm), *Kluyveromyces lactis* (Kl), *Schizosaccharomyces pombe* (Sp), and *Danio rerio* (Dr). Alignment was made based on the structures of *S. cerevisiae* Uba2^ufd^ described herein, and the prior structures of human Uba2 [19], [20]. Sequence identity to *S. cerevisiae* Uba2 is highlighted in magenta, and similarity is highlighted in pink, with residues contacting Ubc9 indicated with a cyan bar. Secondary structures are indicated above. (E) Sequence alignment of *Saccharomyces cerevisiae* Ubc9 sequence (Sc), with the corresponding regions of Ubc9 from human (Hs), *Xenopus laevis* (Xl), *Drosophila melanogaster* (Dm), *Kluyveromyces lactis* (Kl), *Schizosaccharomyces pombe* (Sp), and *Danio rerio* (Dr). Sequence identity to *S. cerevisiae* Ubc9 is highlighted in blue, with residues contacting Uba2^ufd^ indicated with a magenta bar. Secondary structures are indicated above.

To characterize a domain from yeast Uba2 that binds Ubc9, we determined the crystal structure of the isolated yeast Uba2 ubiquitin-fold domain (ufd), corresponding to residues 439–563, at 1.6 Å resolution (**Table 1**, **Figure S1**, **Figure 1**). For the most part, residues 440–551 were clearly visible in the electron density with all secondary structures well-defined and for consistency numbered here according to their counterparts in the prior structures of full-length human Uba2 [19]. Only a loop encompassing residues 531–540 displayed weaker electron density precluding building of several side-chains and resulting in high B-factors, presumably due to greater flexibility.

**Table 1:** Crystallographic and Refinement Statistics.

	Uba2^ufd^	Uba2^ufd^-Ubc9
Accession codes	3ONH.pdb	3ONG.pdb
**Data collection**		
Beamline	ALS 8.2.1	SERCAT ID
Wavelength (Å)	1.00000	1.00000
Space group	I4	P2_1_
**Cell dimensions**		
a, b, c (Å)	a = b = 80.198, c = 50.733	a = 38.465,b = 134.038, c = 62.012
α, β, γ (°)	α = β = γ = 90	α = 90, β = 94.44, γ = 90
Resolution (Å)	50–1.6(1.66–1.6)	45–2.3(2.38–2.30)
Total reflections	289011	334522
Unique reflections	21034	24002
R_merge_ (%)	9.9(58.6)	5.6(26.0)
Average I/σ	17.8(3.0)	28.3(3.5)
Completeness (%)	98.8(96.4)	85.0(53.5)
Redundancy	2.9(2.8)	3.5(2.9)
Wilson B-factor	15.44	48.9
**Refinement**		
Resolution range (Å)	40–1.6	38.4–2.3
No. of reflections (σ≥0)	20193	21393
R_work_ (%)	16.0	22.3
R_free_ (%)	17.8	25.1
Number of protein atoms	885	4267
Number of waters	81	38
Average B-factor (protein)	21	72
Average B-factor (water)	30	61
RMSD:		
Bond lengths (Å)	0.006	0.007
Bond angles (°)	0.98	1.08
Ramachandran plot statistics		
Residues in preferred regions (%)	92.8	90.3
Residues in additional allowed regions (%)	6.3	9.7
Residues in disallowed regions (%)	0.9	0

Highest resolution shell is shown in parenthesis. *R*_work_  =  ∑|*F*_o_–*F*_c_|/∑*F*_o_. *R*_free_ is the cross-validation of *R*-factor, with >5% of the total reflections omitted during model refinement.

Like ubiquitin and Sumo, the Uba2^ufd^ adopts a modified β-grasp fold. Uba2^ufd^ consists of a twisted 5-stranded antiparallel β-sheet on one side, one 3-turn α-helix on the opposite side of the sheet, and two peripheral short helices. The β-sheet and connecting loops form one V-shaped surface. A second V-shaped surface comes from the edge of β-strand 23, the following loop, and α-helix37. The two V-like structures are adjacent to each other, and together form a W-shaped surface (**Figure S2**).

### Structure of a Uba2^ufd^-Ubc9 complex

In order to understand E1-E2 interactions in a Sumo pathway, we determined the crystal structure of a Uba2^ufd^-Ubc9 complex from yeast. Phases were obtained by molecular replacement, using the prior structure of yeast Ubc9 and a polyalanine model of human Uba2 residues 449–546 as searchmodels [19], [40]. There are two molecules per asymmetric unit, which superimpose with 0.3 Å root mean square deviation (rmsd) over all alpha carbons, so only one version of the complex is discussed here. The structure of the Uba2^ufd^ is nearly identical in the complex as on its own (0.47 Å rmsd). The structure of Ubc9 superimposes well with the prior structure of free Ubc9 (0.55 Å rmsd), with the most notable difference being slight variation in the position of the β1β2-loop. The Uba2^ufd^-Ubc9 complex adopts an overall oblong elliptical shape, with the W-shaped surface from Uba2^ufd^ binding the N-terminal α-helix1 and β1β2-loop from Ubc9 (**Figure 1**, **Figure S2**). This interaction is distal - ∼30 Å – to the Ubc9 catalytic cysteine. The Uba2^ufd^-Ubc9 interface buries 1557 Å^2^ of surface area [41].

### Details of Uba2^ufd^-Ubc9 interactions

**Figure 2:**
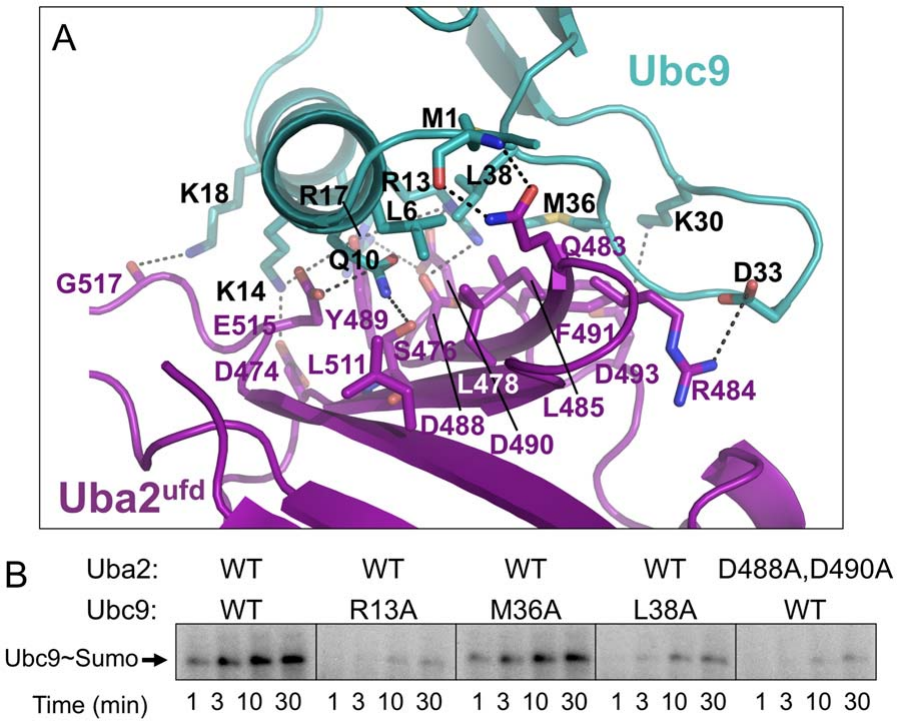
Details of the Uba2^ufd^-Ubc9 interface. (A) Close-up view of the interface between Uba2^ufd^ and Ubc9. Uba2^ufd^ is shown in magenta. Ubc9 is shown in cyan. Nitrogens are colored blue. Oxygens are colored red. Electrostatic interactions are indicated with dashes. (B) Effects of mutations in Uba2 or Ubc9 at the structurally-observed interface. Shown are autoradiograms monitoring ^32^P-labeled yeast Sumo, for time-courses of Aos1-Uba2-catalyzed generation of Ubc9∼ [^32^P]-Sumo thioester conjugate. The wild-type (WT) or mutant version of Uba2 or Ubc9 used in each panel is indicated above.

The Uba2^ufd^-Ubc9 interface is interdigitated, with the two V-shaped grooves from Uba2 binding alternating regions of Ubc9, as in a layered sandwich. The “V” corresponding to the concave Uba2^ufd^ β-sheet holds the Ubc9 N-terminal α-helix1 along its entire length (**Figure 2A**). Ubc9's Leu6 is loosely surrounded by a hydrophobic surface from Uba2's Leu478, Leu485, and Leu511 from three distinct strands. The remainder of the interaction is largely ionic (**Figure 2A**). The Ubc9 N-terminus forms a hydrogen bond with Uba2's Gln483. Ubc9's Gln10 makes a hydrogen bond with Uba2's Ser476 and Glu515. Ubc9's Arg13 makes salt bridges with Uba2's Asp488 and Asp490, and along with Ubc9's Lys14 interacts with Uba2's Tyr489. Ubc9's Lys14 and Arg17 interact with Uba2's Asp474 and Asp490, respectively. At the C-terminus of Ubc9's α-helix1, Lys18 forms a hydrogen bond with the backbone oxygen from Uba2's Gly517.

The Ubc9 β1β2-loop straddles the Uba2 β-strand23. The tip of the Ubc9 β1β2-loop fits in the second “V” shaped groove generated by Uba2 β-strand23, α-helix37, and the intervening loop. Ubc9's Lys30 from one face of the β1β2-loop forms a salt-bridge with Uba2's Asp493. On the other side, Ubc9's Asp33 interacts with Uba2's Arg484. Ubc9's Met36 and Leu38, at the C-terminus of the β1β2-loop, pack against a hydrophobic ridge comprising Uba2's Leu478, Leu485, and Phe491 in the first “V”.

The structure helps explain the deleterious effects of previously reported mutants in yeast Ubc9, which showed that residue substitutions in place of Lys14, or a combination of Lys14, Arg17, and Lys18 from the N-terminal helix, or deleting a portion of the β1β2-loop, hinders binding to full-length Aos1-Uba2 [37]. We further tested the effects of mutating additional key interface residues. Consistent with the structure, individual Ala substitutions in place of Ubc9's Arg13, Met36, or Leu38 hinders Aos1-Uba2-mediated generation of a Ubc9∼Sumo complex (**Figure 2B**, **Figure S3)**. Similar results are observed for mutating the key acidic patch residues Asp488 and 490 from Uba2 (**Figure 2B**, **Figure S3**).

### Conservation of Ubc9 and Uba2 interacting residues across species

Several structural features and individual amino acids involved in the Uba2^ufd^-Ubc9 interaction are conserved among Ubc9s and Uba2s from yeast to humans. Yeast and human Ubc9 share 56% sequence identity (**Figure 1D**). Accordingly, the Uba2-binding side-chains from the Ubc9 α-helix1 are 100% conserved as basic residues. Also, the β1β2-loop structure is conserved among Ubc9s, but is uniquely extended relative to E2s for other UBLs. Here, Asp33 is conserved as acidic, and Met36 and Leu38 are conserved as hydrophobics.

The sequence similarity is reflected by extensive structural similarity between yeast and human Ubc9 (0.53 Å Cα rmsd, previously noted for other structures in [40]). While the many Ubc9 structures, from different organisms and in different complexes, do generally superimpose well, the primary site of very minor differences is in the orientation of the β1β2-loop. It is possible that limited flexibility of this unique Ubc9 structure may play a role in binding to the Uba2 ufd.

**Figure 3:**
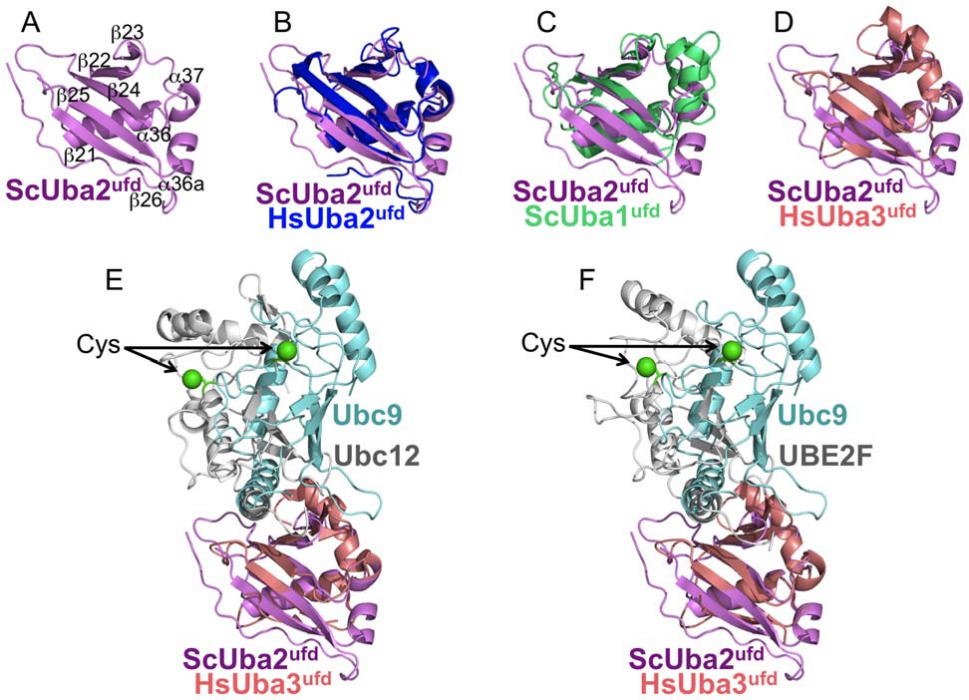
Specificity of E1^ufd^-E2 interactions. (A) Structure of isolated *S. cerevisiae* Uba2^ufd^, shown in magenta. (B) Structure of isolated *S. cerevisiae* Uba2^ufd^ superimposed on the corresponding region of human Uba2 (1Y8Q.pdb, blue) [19]. (C) Structure of isolated *S. cerevisiae* Uba2^ufd^ superimposed on the corresponding region of *S. cerevisiae* Uba1, the E1 for ubiquitin (3CMM.pdb, green) [28]. (D) Structure of isolated *S. cerevisiae* Uba2^ufd^ superimposed on the corresponding region of human Uba3 (1Y8X.pdb, peach) [31]. (E) Comparison of E1-E2 interactions in Sumo and NEDD8 pathways by superimposing the E1 ufds from the Uba2^ufd^–Ubc9 complex structure with a prior human Uba3^ufd^-Ubc12 (grey) structure (1Y8X.pdb) [31]. (F) Comparison of E1-E2 interactions in Sumo and NEDD8 pathways by superimposing the E1 ufds from the Uba2^ufd^–Ubc9 complex structure with a prior human Uba3^ufd^-UBE2F (grey) structure (3FN1.pdb) [33].

The sequences between Uba2 ufds are generally less conserved (**Figure 1E**, **Figure 3A**, **Figure 3B**). Despite only 17% sequence identity, a search of the Protein Data Bank using DALI identifies the ufd region from a prior structure of full-length human Uba2 (also called Sae2) as having the highest degree of structural similarity with the yeast Uba2^ufd^ (Cα rmsd 1.85 Å) [19], [20], [42]. The main differences are localized to two regions, which are distal to the Ubc9-binding site. The first difference is the short α-helix36a, which corresponds to an insertion in the sequence of yeast Uba2 between β-strand21 and α-helix 36 (**Figure 1E**, **Figure 3A**, **Figure 3B**). The second is at the C-terminal portion of the domain where the sequences are most divergent: the loop preceding β-strand24, the loop between β-strands24 and 25, and the region following β-strand25 that is disordered in human Uba2 but in the yeast Uba2^ufd^ is ordered and forms a more extended structure. Nonetheless, the striking similarity of the overall structures of yeast and human Uba2 ufds supports the notion that the structurally observed yeast Uba2^ufd^-Ubc9 interactions will be preserved across species. Indeed, some key interface residues are relatively conserved, including Ser476 (Ser or Asp across species), Arg484 (charged or polar), Leu485 (Leu, Ile, or Val across species), and Asp488 and Asp490 (Ser or Asp, and Asp or Glu across species, respectively) (**Figure 1E**). Furthermore, human Ubc9 was shown to cause chemical shift perturbations in the corresponding region of human Uba2^ufd^ [36].

### Comparison of Uba2^ufd^-Ubc9 with prior E1ufd structures from other UBL pathways

To gain insights into similarities and differences among E1 ubiquitin-fold domains, we compared the yeast Uba2^ufd^–Ubc9 crystal structure with the corresponding regions of crystal structures of yeast Uba1 (E1 for ubiquitin) and human Uba3 (E1 for NEDD8) (**Figure 3A**, **Figure 3C**, **Figure 3D**, **Figure S4**). The yeast Uba2^ufd^ displays overall structural similarity to the ufds from Uba1 and Uba3 (2.4 and 1.6 Å rmsd, respectively), including the W-shaped surface. Furthermore, structural comparison of Uba2^ufd^-Ubc9 with prior structures of Uba3 in complex with NEDD8 E2s (Ubc12 or UBE2F) reveal that the Sumo and NEDD8 pathways use parallel E1^ufd^ and E2 surfaces to mediate interactions (**Figure 3E**, **Figure 3F**).

Despite their overall common mode of binding, an intriguing difference is that the relative positions of the E1^ufd^ and E2 catalytic cysteines are considerably offset between Uba2^ufd^–Ubc9 and Uba3^ufd^–Ubc12 or Uba3^ufd^–UBE2F (**Figure 3E**, **Figure 3F**). The detailed interactions between Uba2 and Ubc9 described above (**Figure 2A**) also differ substantially from corresponding contacts in the NEDD8 pathway [31], [33]. Furthermore, global structural differences between Uba2 and other E1 ufds, and between Ubc9 and other E2s help explain why Ubc9 does not bind E1s for other UBLs and vice-versa. In the Uba1 ufd the region corresponding to the Uba2^ufd^'s short α-helix37 is extended by a complete turn, and the preceding loop in Uba2^ufd^ corresponds to a 2-turn helix in Uba1 (**Figure 3C**) [28]. This latter helix is even longer in Uba3's ufd (**Figure 3D**) [27], [31]. These Uba1 and UBA3 helical insertions would clash with the Uba2-binding basic residues from Ubc9's α-helix1 and extended β1β2 loop. Thus, several structural differences between E1^ufd^s, and corresponding distinctions between their cognate E2s, may help establish specificity of E1-E2 interactions.

### Docking Uba2^ufd^-Ubc9 onto prior full-length human Aos1-Uba2 structures

**Figure 4:**
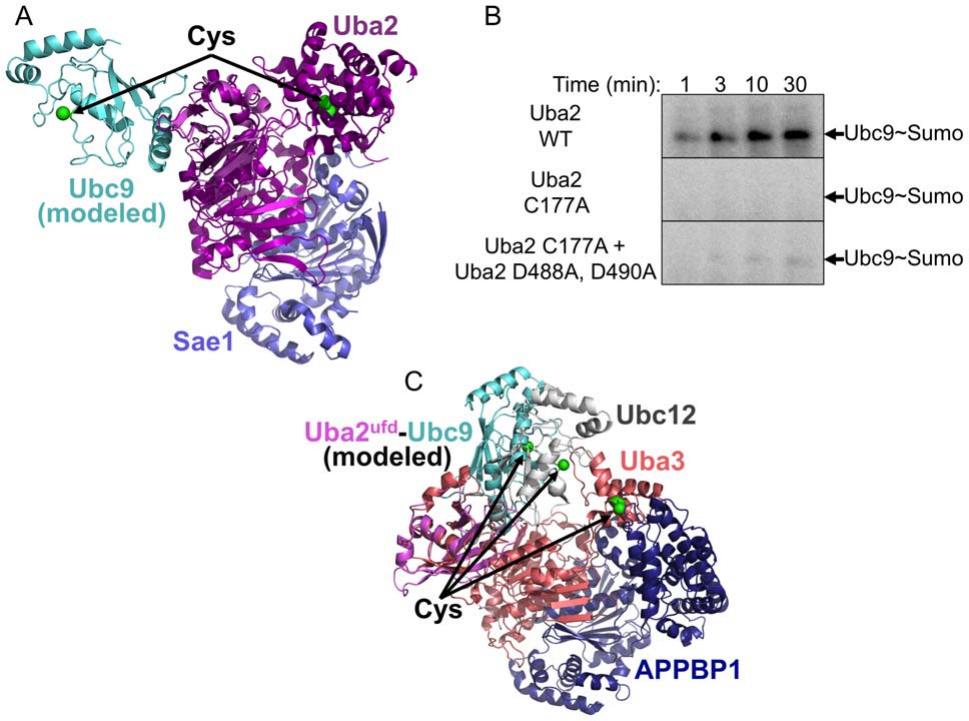
Implications of Uba2^ufd^–Ubc9 structure for Smt3 transfer from Uba2 to Ubc9. (A) Structure of *S. cerevisiae* Uba2^ufd^–Ubc9 (magenta, cyan), with Uba2^ufd^ superimposed on the corresponding region of human Uba2 from the Sae1-Uba2-Sumo1∼AMSN complex (3KYC.pdb) [20]. Sae1 is shown in slate, human Uba2 is shown in purple, and Sumo∼AMSN is not shown for simplification. Catalytic cysteine residues of Ubc9 and Uba2 are shown in green. (B) Autoradiograms monitoring ^32^P-labeled Sumo, for time-courses of Aos1-Uba2-catalyzed generation of Ubc9∼ [^32^P]-Sumo thioester conjugate. WT – wild-type; Uba2 C177A – cysteine-to-alanine mutation at Uba2 catalytic Cys177. Uba2 D488A,D490A – aspartate-to-alanine mutation at Uba2's Ubc9-interacting Asp488 and Asp490. (C) Structure of *S. cerevisiae* Uba2^ufd^–Ubc9 (magenta, cyan), with Uba2^ufd^ superimposed on the corresponding region of human Uba3 (peach) from the APPBP1 (blue) -Uba3∼NEDD8-NEDD8-Ubc12(C/A)-MgATP complex [32]. For simplification, the NEDD8 molecules are not shown, and the E2 active site positions (an Ala mutation in Ubc12) are labeled with green spheres, and “Cys” to denote their locations.

In order to gain insights into Sumo transfer from Uba2 to Ubc9, we superimposed the Uba2^ufd^-Ubc9 structure onto the corresponding region of human Uba2 from several recent crystal structures [19], [20]. The overall locations of domains, including the ufd, are generally similar in structures of human Aos1-Uba2-MgATP (also called Sae1-Sae2-MgATP, 1Y8Q.pdb), Aos1-Uba2-Sumo-MgATP (1Y8R.pdb), and Aos1-Uba2-Sumo∼AMSN (a chemical modification mimicking Sumo C-terminal adenylation, 3KYC.pdb) [19], [20]. The ufd is also in a similar relative orientation in a recent structure of human Aos1-Uba2∼Sumo∼AVSN, which mimics the tetrahedral intermediate during Uba2's catalytic cysteine's attack on the C-terminally adenylated Sumo (3KYD.pdb), so only the comparison to Aos1-Uba2-Sumo∼AMSN is shown in **Figure 4A**. All the structures of full-length Uba2 lack Ubc9, and represent states of Aos1-Uba2 prior to Sumo transfer to Ubc9 [19]. All docking models on full-length Uba2 would place Ubc9's catalytic cysteine distal to and facing the opposite direction of Uba2's catalytic cysteine (**Figure 4A**).

To address the possibility of whether Sumo thioester-linked to the catalytic Cys177 of one Uba2 can be transferred to Ubc9 bound to another Uba2, we examined generation of the Ubc9∼Sumo thioester-linked conjugate upon mixing two defective mutant versions of the Sumo E1. In one mutant, the Uba2 catalytic Cys177 was mutated to Ala to prevent formation of a Uba2∼Sumo intermediate, but the ufd was wild-type. The other retained ability to form a thioester linkage with Sumo at Cys177, but harbored Ala mutations in the Ubc9-binding ufd residues Asp488 and Asp490. On their own, each mutant is defective for generating the thioester-linked Ubc9∼Sumo conjugate. Mixing the two E1s does not rescue the other's defect (**Figure 4B**).

Thus, it seems likely that Uba2 undergoes a conformational change to bring the Uba2 and Ubc9 catalytic cysteines together for Sumo transfer. Previous structural studies of the NEDD8 E1, which showed dramatic ufd reorientation, may provide some insights into such a conformational change. Superimposing Uba2^ufd^-Ubc9 on the Uba3^ufd^ in a structure of the NEDD8 E1 complexed with two molecules of NEDD8 and a catalytically-inactive E2 (Ubc12), which represents an intermediate en route to UBL transfer from E1 to E2, shows how ufd rotation might allow Ubc9 to be reoriented toward Uba2's catalytic cysteine [32]. However, even upon rotation of the Uba2 ufd to the position observed in the NEDD8 E1, a significant E1-to-E2 cysteine-to-cysteine gap remains (**Figure 4C**). It is possible that Uba2's ufd undergoes an even greater rotation during Sumo transfer. Another excellent possibility for how the gap between the Uba2 and Ubc9 catalytic cysteines could be closed comes from the recent landmark finding that the Uba2 catalytic cysteine domain can undergo dramatic reorientation and remodeling [20]. It seems likely that the proposed combination of ufd and catalytic cysteine domain rotations bring the Uba2 and Ubc9 cysteines together.

### Implications for E1-E2-E3 cascades in the Sumo pathway

**Figure 5:**
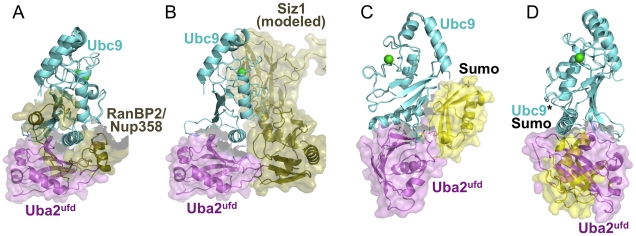
Implications of Uba2^ufd^–Ubc9 structure for Sumo cascades. (A) Comparison of E1-E2 and E2-E3 interactions in Sumo cascades. Structure of *S. cerevisiae* Uba2^ufd^–Ubc9 (magenta, cyan), with Ubc9 superimposed on the previous structure of human Ubc9 (cyan) bound to RanBP2/Nup358 (olive) from the complex with RanGAP-1 (not shown) and Sumo-1 (not shown) [24]. Uba2^ufd^ and a portion of RanBP2/Nup358 are shown with a semi-transparent surface to highlight the overlapping regions. (B) Structure of *S. cerevisiae* Uba2^ufd^–Ubc9 (magenta, cyan), with Ubc9 superimposed on a model for *S. cerevisiae* Ubc9-Siz1 (olive), based on previous structures of Siz1 and c-Cbl-UbcH7 [25], [46]. Briefly, the RING domain c-Cbl and UbcH7 were docked onto the SP-RING domain of Siz1, and then Ubc9 was modeled in place of UbcH7. Uba2^ufd^ and Siz1 are shown with a semi-transparent surface to highlight the overlapping regions. (C) Comparison of E1-E2 and noncovalent E2-UBL interactions in Sumo cascades. Structure of *S. cerevisiae* Uba2^ufd^–Ubc9 (magenta, cyan) superimposed in previous structure of noncovalent Ubc9-Sumo (cyan, yellow) complex from *S. cerevisiae* [47]. Uba2^ufd^ and Sumo are shown with a semi-transparent surface to highlight the overlapping regions. (D) Structure of *S. cerevisiae* Uba2^ufd^–Ubc9 (magenta, cyan), with Ubc9 superimposed on human Ubc9*Sumo (cyan*yellow) [50]. Uba2^ufd^ and Sumo are shown with a semi-transparent surface to highlight the overlapping regions.

E2s play the central role in UBL cascades. Thus, after receiving Sumo from Uba2, Ubc9 ultimately transfers Sumo to a target. This is often facilitated by an E3. Two types of Sumo E3s have been structurally characterized: (1) a neither-HECT-nor-RING E3, as in RanBP2/Nup358, which binds both Ubc9 and its covalently-linked Sumo to optimally orient the thioester bond for Sumo transfer to a substrate Lys [24], [43], [44], and (2) Siz/PIAS E3s, which contain a SP-RING domain that structurally resembles RING E3s utilized by the ubiquitin and NEDD8 pathway [25], [45]. To gain insights into how Sumo E1-E2-E3-target cascades are organized, we superimposed Ubc9 from our Uba2^ufd^-Ubc9 structure with that in the prior crystal structure of Sumo∼RanGAP1-Ubc9-RanBP2/Nup358 [24], and with a structural model of Ubc9-Siz1 based on other E2-RING domain structures [25], [46]. Uba2^ufd^ and RanBP2/Nup358 bind overlapping surfaces on Ubc9 [24] (**Figure 5A**). Structural modeling also indicates that Uba2^ufd^ would clash with the Siz1 SP-CTD domain, which is C-terminal of the SP-RING domain and is conserved among Siz/PIAS E3s (**Figure 5B**) [25]. Thus, Uba2-mediated formation of a Ubc9∼Sumo thioester conjugate may be mutually exclusive with E3-mediated Sumo ligation to targets.

Sumo also binds noncovalently to Ubc9, via the “backside” distal from the E2 catalytic cysteine [47], [48], [49]. This interaction is conserved from yeast to humans [47], [48], [49]. Although the function of this noncovalent Ubc9-Sumo interaction is not well understood, mutant versions of Ubc9 where this interaction is disrupted cannot support yeast viability [47], and are impaired at generating long poly-Sumo chains [48], [49]. Previous biochemical data showed that the noncovalent Sumo interaction competes with Ubc9's interaction with Uba2 [37], [47]. Indeed, superposition of Ubc9 from our complex with Uba2^ufd^ with prior structures of complexes with Sumo reveal mutually exclusive binding to Ubc9's α-helix1, particularly Arg13 and Arg17 (**Figure 5C**). Thus, the Uba2^ufd^ recognizes multipurpose binding sites on Ubc9, which may serve to order association with many different partners in the Sumo cascade.

In a related vein, human Ubc9 is distinct from yeast Ubc9 in becoming modified on Lys14 via an isopeptide bond to human Sumo, forming a covalent complex referred to as Ubc9*Sumo [50]. Ubc9*Sumo can bind E1 and form a thioester-linked complex to another Sumo at its active site, which subsequently can be transferred to targets. Sumoylation of Ubc9 shifts its E3-independent target specificity [50]. For the substrate Sp100, it seems Sp100's Sumo-interacting motif (SIM) is recruited to the Sumo linked to Ubc9's Lys14. Comparison of the crystal structure of human Ubc9*Sumo with that of yeast Uba2^ufd^-Ubc9 reveals that Uba2 overlaps the position of Sumo in the covalent complex (**Figure 5D**). How then might human Ubc9*Sumo bind Uba2 to become charged with Sumo? Given the high structural similarity between human and yeast Ubc9 [40] and Uba2^ufd^ (**Figure 3**), and prior NMR and mutational data consistent with conservation of our structurally-observed Uba2^ufd^-Ubc9 interactions in their human counterparts [19], [36], [51], it seems likely that human Uba2 and Ubc9 interact as in our structure. A plausible model would be that there is some flexibility between Ubc9 and Sumo in Ubc9*Sumo such that Sumo's β-grasp domain moves out of the way when Uba2 binds. This is consistent with ∼4-fold more human Aos1-Uba2 being required to fully bind Ubc9*Sumo in comparison to Ubc9 in a qualitative interaction study [50]. Additional interactions might stabilize Uba2-Ubc9 complex formation during the transthiolation reaction. Indeed, although quantitative enzyme kinetic comparison was not performed, under some conditions Ubc9 and Ubc9*Sumo show equal formation of thioester-linked intermediates with Sumo [50]. Future studies will be required to understand the dynamics of interactions between Sumo, Ubc9, Uba2, and E3s in transfer cascades.

### Conclusions

The structure of the yeast Uba2^ufd^ in complex with Ubc9 provides the first crystallographic insights into E1-E2 contacts in a Sumo pathway. The structure is consistent with mutational data presented previously and herein, which validate these interactions for Sumo conjugation to Ubc9 [19], [30], [36], [37], [40], [51].

The complex structure also provides only the second detailed view of E1^ufd^-E2 interactions for any UBL, and demonstrates a common overall mode of E2 recruitment by E1 ufds: a ufd's “W” (i.e., double-“V”) -shaped surface engages an E2's N-terminal helix and β1β2-loop. Specificity within the Sumo pathway is established by many unique favorable interactions between Uba2 and Ubc9. Furthermore, Ubc9 is distinct among E2s as having an extended rigid β1β2-loop. This is recognized by a conserved C-terminal “V” from Uba2^ufd^, comprised of Uba2 elements spanning from β-strand23 through α-helix37. The Uba2^ufd^ C-terminal “V” is minimized relative to the corresponding regions in ubiquitin and NEDD8 E1s [27], [28], which would be unable to accommodate the Ubc9 β1β2-loop. Structural comparison to Ubc9-E3 structures/models, and to Ubc9-Sumo, demonstrates how E1-E2 interactions are mutually exclusive with forming E2-E3 or noncovalent E2-UBL complexes in Sumo cascades.

Finally, docking the Uba2^ufd^-Ubc9 structure on prior structures of full-length Uba2 indicate that the Uba2 ufd would need to undergo significant rotation so that the Uba2 and Ubc9 cysteines could face each other. This is also a common feature of previous structures from the NEDD8 cascade [27], [31], [32], [52]. Furthermore, based on conformational changes observed for Uba2 at earlier steps in the Sumo activation cycle, it seems that the Uba2 cysteine domain may also undergo conformational changes to meet the Ubc9 cysteine for Sumo transfer [20]. Along these lines, it is noteworthy that unique features to Uba2s and Ubc9s also establish a distinct orientation of the E2 catalytic cysteine relative to the E1's E2-binding ufd (**Figure 3E**, **Figure 3F**). It will be interesting in the future to understand how UBL-specific E1 conformational changes accommodate such distinct geometries during E1-to-E2 UBL transfer.

## Materials and Methods

### Cloning, protein expression and purification

Expression constructs were made by standard PCR/ligation procedures, with sequences verified by automated sequencing procedures. Yeast Sumo, Smt3, is referred to as Sumo in the text. For biochemical studies, Smt3/Sumo residues 13–97 were expressed from pGEX-2TK (GE), harboring an N-terminal site for phosphorylation by Protein Kinase A (PKA), and was purified as previously described for other UBLs [33].

Uba2^ufd^ (residues 439–563) was expressed from pGEX-4T1 (GE). BL21(DE3) harboring the expression construct were grown in LB with ampicillin (100 µg/mL) at 37°C until reaching an OD_600_ of ∼1.0. Expression was induced by the addition of IPTG to a final concentration of 0.6 mM, followed by overnight culturing at room temperature. Cells were resuspended in 50 mM Tris-HCl, 0.2 M NaCl, 5 mM DTT, pH 8.0 supplemented with 2.5 mM with PMSF, and lysed by sonication on ice. GST-Uba2^ufd^ was purified by glutathione-affinity chromatography, treated with a 1∶100 ratio of thrombin during overnight dialysis into 25 mM Tris-HCl, 0.2 M NaCl, 5 mM DTT, pH 8.0 at 4°C overnight. GST and any remaining GST-Uba2^ufd^ fusion protein were removed by glutathione-affinity. For co-crystallization, Uba2^ufd^ was purified by gel filtration chromatography using a SD75 column (GE) in 25 mM Tris-HCl, 0.15 M NaCl, 5 mM DTT, pH 7.6, concentrated to 22 mg/ml (Bio-Rad Protein Assay), aliquotted, flash-frozen in liquid nitrogen, and stored at −80°C until further use. For crystallization on its own, Uba2^ufd^ prepared similarly, except that gel filtration was performed in 50 mM Tris-HCl, 0.2 M NaCl, 5 mM DTT, pH 7.6, and Uba2^ufd^ was concentrated to 40 mg/ml.

Ubc9 was expressed from pGEX4T3 (GE) [37] in BL21(DE3) Gold cells. After growth at 37°C until the OD_660_ reached ∼0.8, expression was induced by the addition of 1 mL of 0.6 M IPTG/liter of culture, and induction was carried out overnight at 16°C. GST-Ubc9 was purified by glutathione affinity. For biochemical studies, GST-Ubc9 bound to glutathione sepharose was treated with thrombin overnight at 4°C to release wild-type and mutant versions of Ubc9. Wild-type and mutant versions of Ubc9 were concentrated in a final buffer of 50 mM Tris-HCl pH 7.6, 200 mM NaCl, 1 mM DTT, aliquotted, flash-frozen, and stored until use. For crystallography, GST-Ubc9 was treated with a 1∶30 ratio of thrombin overnight at 16°C. Ubc9 was purified to homogeneity by cation exchange with a homemade column packed with ResourceS resin (GE) using a NaCl gradient in 20 mM HEPES, 1 mM DTT, pH 7.0, concentrated to 27 mg/ml, aliquotted, flash-frozen in liquid nitrogen, and stored at −80°C until further use.

For biochemical assays, the expression construct for Sumo E1 (the heterodimeric Aos1-Uba2 complex) was described previously [37]. BL21(DE3) Gold cells harboring the expression construct were grown in LB with ampicillin (100 µg/mL) at 37°C until reaching an OD_600_ of ∼0.8. After cooling to 16°C for 45 minutes, expression was induced by the addition of 1 mL of 0.2 M IPTG/liter of culture, shaking overnight at 16°C. GST-Aos1-Uba2 was purified by glutathione affinity chromatography, and eluted protein was cleaved overnight with thrombin at 4°C. Aos1-Uba2 was further purified by anion exchange chromatography, concentrated in a final buffer of 50 mM Tris-HCl pH 7.6, ∼290 mM NaCl, 1 mM DTT, aliquotted, flash-frozen in liquid nitrogen, and stored at −80°C until further use.

### Crystallization, data collection, and structure determination and refinement

Initial crystallization screening was performed with a Mosquito crystallization robot (TTP Labtech) with commercial 96-well screens. After testing several conditions (chemical and temperature) and Uba2^ufd^-Ubc9 complexes (from human and yeast with different ufd domain boundaries), crystals were obtained at 4°C in the Index HTS condition G2 (Hampton Research), for a 1.1∶1 volume:volume mixture of Uba2^ufd^ at 22 mg/ml and Ubc9 at 27 mg/ml. A diffraction-quality crystal was obtained after multiple cycles of streak-seeding into 20% (w/v) PEG3350, 0.1 M Bis-Tris pH 5.5, 0.2 M Li_2_SO_4_, and harvested and flash-frozen in mother liquor supplemented with 20% (v/v) glycerol. The crystals belong to space-group P2_1_. Data to 2.3 Å resolution were obtained at the SERCAT-ID beamline, and processed with HKL2000 [53]. Initial models for the complex were obtained by performing molecular replacement in the P1 space group using PHASER [54], after searching for 4 copies of the structure of yeast Ubc9 (2GJD.pdb, Chain A) [40] and of a polyAla model of human Uba2 residues 449–546 from 1Y8Q.pdb, Chain C [19]. One of the 4 complexes from this solution was subsequently used as a search model to find two Uba2^ufd^–Ubc9 complexes per asymmetric unit in P2_1_. The model was built using Coot [55] and refined using TLS parameters in REFMAC [56]. Due to low sequence similarity with the human Uba2^ufd^, we wished to obtain additional data to confirm the structure in regions where side-chains were poorly visible. We thus obtained crystals for the isolated Uba2^ufd^ at 4°C in the Ammonium Sulfate HTS condition H8 (Qiagen). After optimization in manual trays, a crystal was obtained in 3 M (NH_4_)_2_SO_4_, 1% (v/v) MPD, and harvested and flash-frozen in 3 M AmSO4, 1% (v/v) MPD, 25% (v/v) glycerol. Data to 1.6 Å resolution were obtained at the SERCAT-ID beamline, and processed with HKL2000 [53]. Examination of the data with Phenix.xtriage indicated the space group to be I_4_, with nearly perfect merohedral twinning and a twin operator of -k,-h,-l. The structure was obtained by molecular replacement using PHASER [54], with the Uba2^ufd^ from the partially-refined Uba2^ufd^-Ubc9 complex structure as a search model for one molecule in the asymmetric unit. After rebuilding using Coot [55] and refinement including twin laws using Phenix [57], the isolated Uba2^ufd^ structure was then used as a guide for final refinement of the Uba2^ufd^-Ubc9 structure. Data collection and refinement statistics are provided in **Table 1**. The single Ramachandran outlier in the high resolution Uba2^ufd^ structure corresponds to Gly517, with the conformation supported by the electron density.

### Assays for formation of a Ubc9∼Sumo thioester conjugate

Sumo was labeled with ^32^P at an N-terminal PKA site using PKA (New England Biolabs) and [γ-^32^P]ATP as described previously for other UBLs [33]. Reactions were performed at 18°C, which is the ambient room temperature for our dedicated room for use of radioactivity, in 10 µl reaction volumes containing 50 mM Tris-HCl (pH 7.6), 5 mM ATP, 10 mM MgCl_2_, 2 mg/ml BSA, 5 mM creatine phosphate, 0.3 U/ml creatine kinase, 0.3 U/ml inorganic pyrophosphatase, 5 nM Aos1-Uba2, 250 nM Ubc9 or the indicated mutant, and 10 µM [γ-^32^P]-Sumo. Reactions were quenched at 1, 3, 10 and 30 minutes by the addition of an equal volume of 2X SDS sample buffer, resolved on 15% SDS-PAGE gels, dried, and exposed to film. In the assay containing both Aos1-Uba2 (Cys177Ala) and Aos1-Uba2 (Asp488Ala and Asp490Ala), 5 nM of both mutant E1s was used.

## Supporting Information

Figure S1 **Representative electron density.** (A) Final 2F_o_–F_c_ electron density contoured at 1.4σ (green mesh) is shown over Uba2^ufd^ (magenta) – Ubc9 (cyan) complex. (B) Final 2F_o_–F_c_ electron density contoured at 1.4σ (blue mesh) is shown over Uba2^ufd^ structure.
(TIF)

Figure S2 **Ubc9 binds W-shaped groove in Uba2^ufd^.** Cartoon view of the overall structure of the complex, with Uba2^ufd^ is shown in magenta surface and Ubc9 is shown in cyan. The W-shaped groove in Uba2^ufd^ is indicated.
(TIF)

Figure S3 **Effects of mutations in either yeast Uba2 or Ubc9 on forming the thioester-linked Ubc9∼Sumo thioester conjugate.** (A) Amount of Ubc9∼ [^32^P]Sumo thioester formed over time. Counts were normalized by comparison to the amount of the Ubc9∼ [^32^P]Sumo reaction product generated for wild-type Uba2 and Ubc9 enzymes at 30 minutes. Error bars represent standard error from experiments performed three independent times. (B) 30-minute time points for reactions shown in A, but treated with DTT prior to SDS-PAGE.
(TIF)

Figure S4 **Sequence comparisons of Ubc9, Uba2^ufd^, and corresponding regions of E2s and E1s for other UBLs.** (A) Sequence alignment of *Saccharomyces cerevisiae* Ubc9 sequence (Sc), with Ubc9 from human (Hs), and the catalytic core domain regions of the human E2s for NEDD8 (Ubc12 and UBE2F). Sequences were aligned based on structures. Secondary structures are indicated above. (B) Sequence alignment of *Saccharomyces cerevisiae* Uba2^ufd^ sequence (Sc), with the corresponding regions of Uba2 from human (Hs), and the ufds from E1s for ubiquitin (Uba1) and NEDD8 (Uba3). Sequences were aligned based on structures. Secondary structures from the Sc Uba2^ufd^ structure are indicated above.
(TIF)
