# Supplementary figures and images for: Crystal Structure of UBA2ufd-Ubc9: Insights into E1-E2 Interactions in Sumo Pathways
Source: PLoS One. 2010 Dec 30;5(12):e15805. doi: 10.1371/journal.pone.0015805 (PMC3012696; doi:10.1371/journal.pone.0015805)

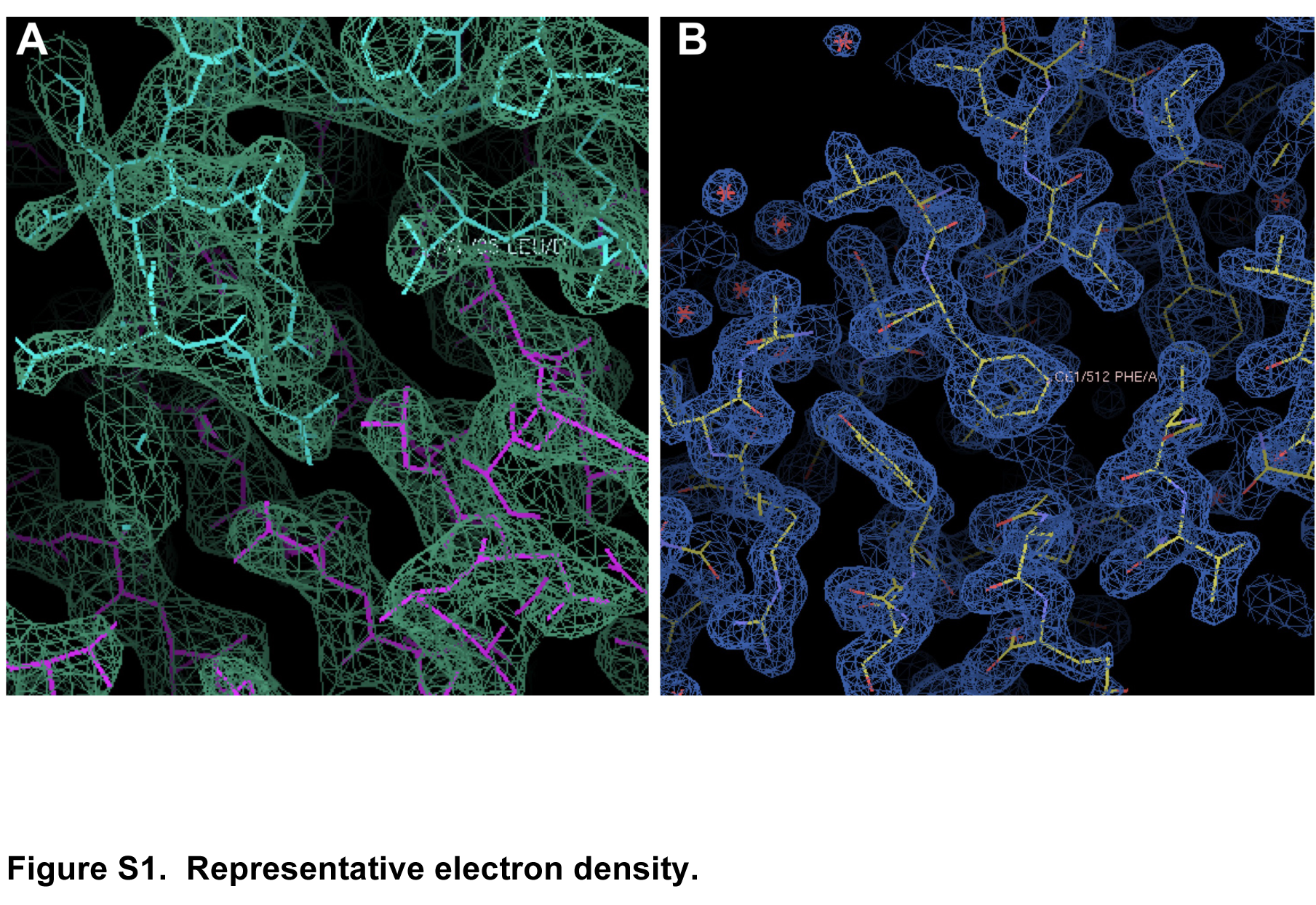

Supplement: Figure S1 — Representative electron density. (A) Final 2Fo–Fc electron density contoured at 1.4σ (green mesh) is shown over Uba2ufd (magenta) – Ubc9 (cyan) complex. (B) Final 2Fo–Fc electron density contoured at 1.4σ (blue mesh) is shown over Uba2ufd structure. (TIF) [file pone.0015805.s001.tif]

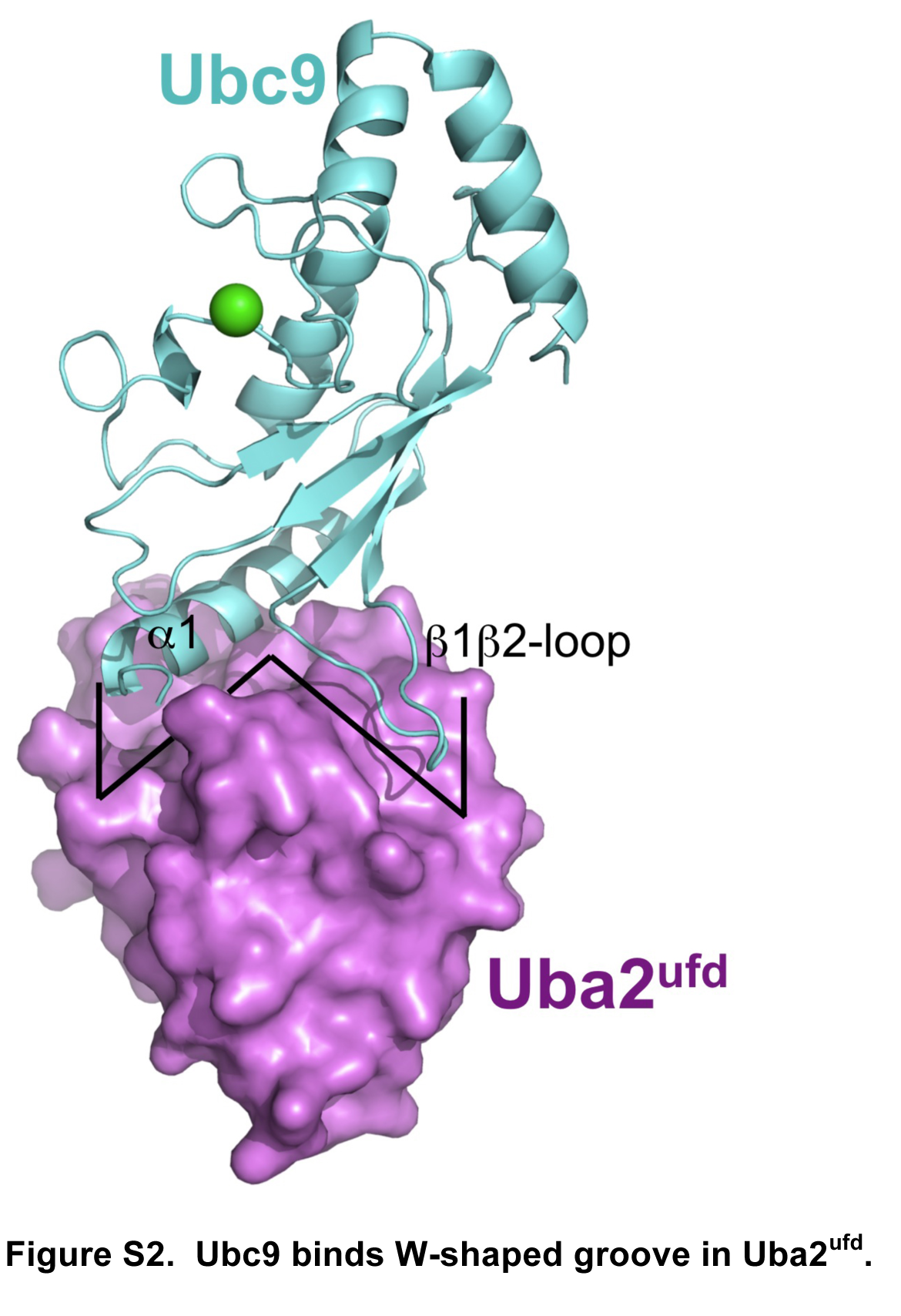

Supplement: Figure S2 — Ubc9 binds W-shaped groove in Uba2ufd. Cartoon view of the overall structure of the complex, with Uba2ufd is shown in magenta surface and Ubc9 is shown in cyan. The W-shaped groove in Uba2ufd is indicated. (TIF) [file pone.0015805.s002.tif]

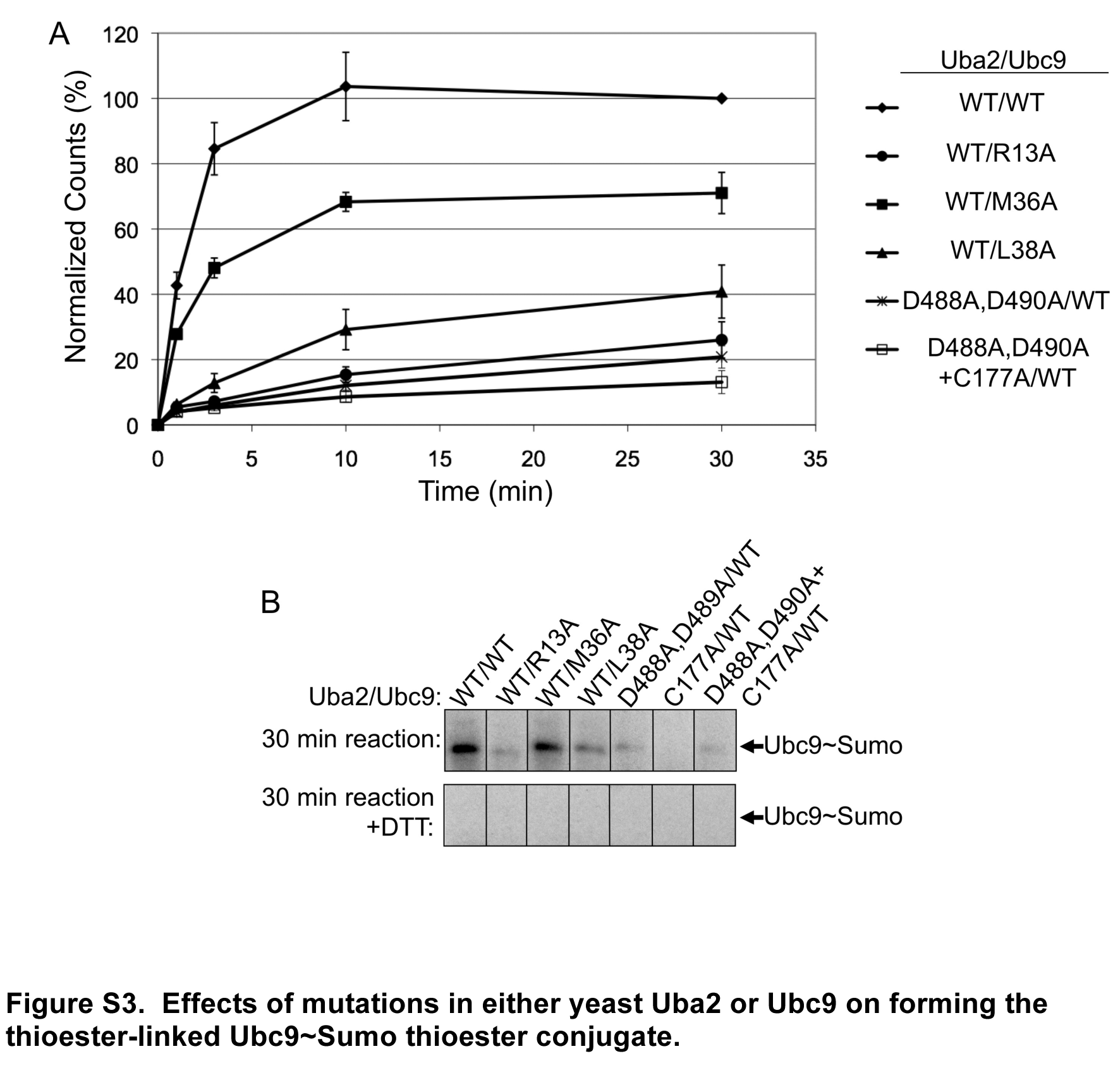

Supplement: Figure S3 — Effects of mutations in either yeast Uba2 or Ubc9 on forming the thioester-linked Ubc9∼Sumo thioester conjugate. (A) Amount of Ubc9∼ [32P]Sumo thioester formed over time. Counts were normalized by comparison to the amount of the Ubc9∼ [32P]Sumo reaction product generated for wild-type Uba2 and Ubc9 enzymes at 30 minutes. Error bars represent standard error from experiments performed three independent times. (B) 30-minute time points for reactions shown in A, but treated with DTT prior to SDS-PAGE. (TIF) [file pone.0015805.s003.tif]

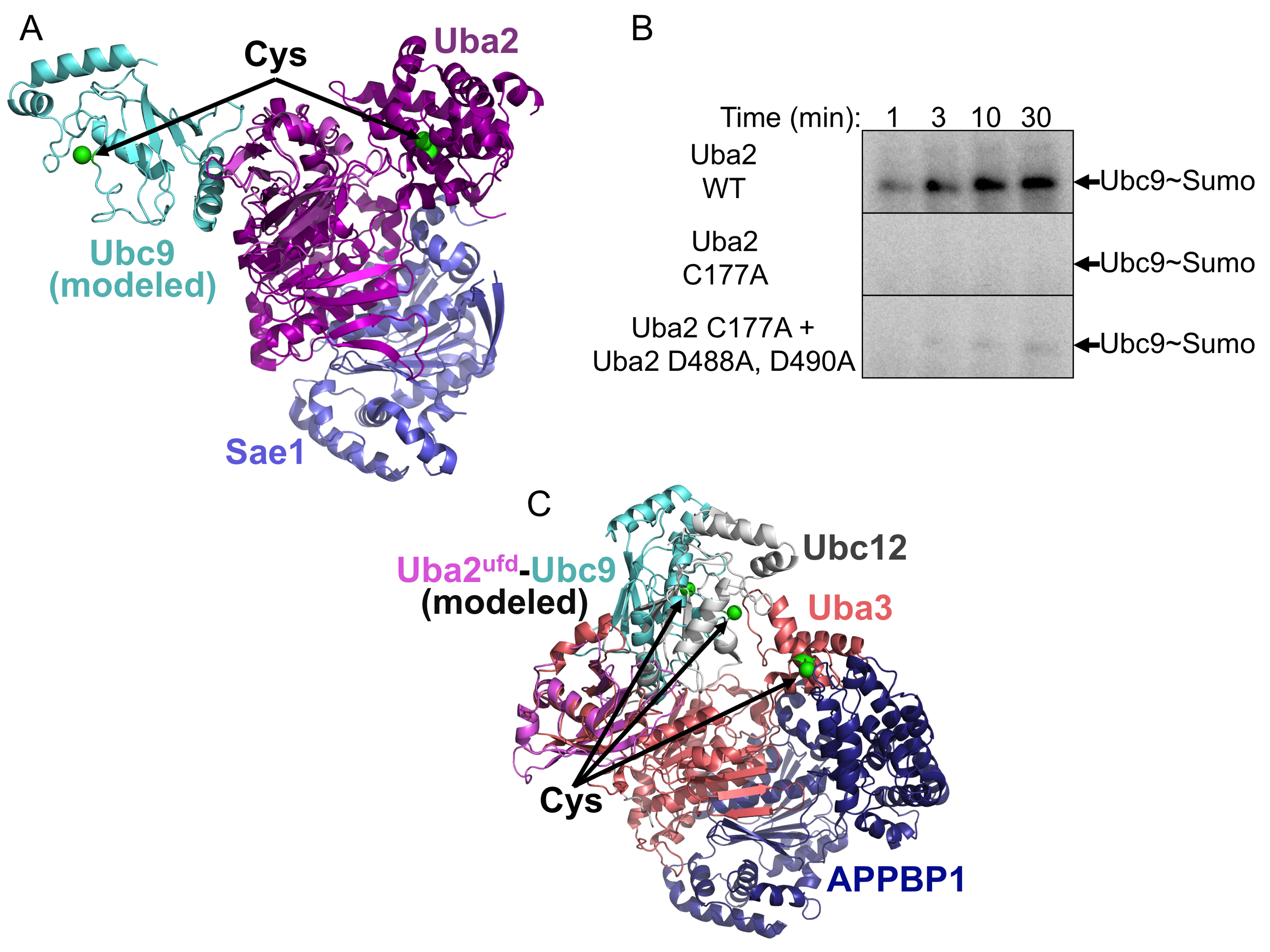

Supplement: Figure S4 — Sequence comparisons of Ubc9, Uba2ufd, and corresponding regions of E2s and E1s for other UBLs. (A) Sequence alignment of Saccharomyces cerevisiae Ubc9 sequence (Sc), with Ubc9 from human (Hs), and the catalytic core domain regions of the human E2s for NEDD8 (Ubc12 and UBE2F). Sequences were aligned based on structures. Secondary structures are indicated above. (B) Sequence alignment of Saccharomyces cerevisiae Uba2ufd sequence (Sc), with the corresponding regions of Uba2 from human (Hs), and the ufds from E1s for ubiquitin (Uba1) and NEDD8 (Uba3). Sequences were aligned based on structures. Secondary structures from the Sc Uba2ufd structure are indicated above. (TIF) [file pone.0015805.s004.tif]
